# BK Polyomavirus Activates HSF1 Stimulating Human Kidney Hek293 Cell Proliferation

**DOI:** 10.1155/2021/9176993

**Published:** 2021-11-20

**Authors:** Sara Baldelli, Dolores Limongi, Cristiana Coni, Fabio Ciccarone, Marco Ciotti, Paola Checconi, Anna Teresa Palamara, Maria Rosa Ciriolo

**Affiliations:** ^1^IRCCS San Raffaele Pisana, Department of Human Sciences and Promotion of the Quality of Life, San Raffaele Roma Open University, Rome, Italy; ^2^IRCCS San Raffaele “La Pisana”, Rome, Italy; ^3^Laboratory of Molecular Virology, Polyclinic Tor Vergata Foundation, Rome, Italy; ^4^Department of Public Health and Infectious Diseases, Sapienza University of Rome, Laboratory Affiliated to Istituto Pasteur Italia-Fondazione Cenci Bolognetti, Rome, Italy; ^5^Department of Biology, University of Rome “Tor Vergata”, Rome, Italy

## Abstract

**Objectives:**

Some DNA viruses, such as BKPyV, are capable of inducing neoplastic transformation in human tissues through still unclear mechanisms. The goal of this study is to investigate the carcinogenic potential of BK polyomavirus (BKPyV) in human embryonic kidney 293 (Hek293) cells, dissecting the molecular mechanism that determines the neoplastic transformation.

**Materials and Methods:**

BKPyV, isolated from urine samples of infected patients, was used to infect monolayers of Hek293 cells. Subsequently, intracellular redox changes, GSH/GSSH concentration by HPLC, and reactive oxygen/nitrogen species (ROS/RNS) production were monitored. Moreover, to understand the signaling pathway underlying the neoplastic transformation, the redox-sensitive HFS1-Hsp27 molecular axis was examined using the flavonoid quercetin and polishort hairpin RNA technologies.

**Results:**

The data obtained show that while BKPyV replication is closely linked to the transcription factor p53, the increase in Hek293 cell proliferation is due to the activation of the signaling pathway mediated by HSF1-Hsp27. In fact, its inhibition blocks viral replication and cell growth, respectively.

**Conclusions:**

The HSF1-Hsp27 signaling pathway is involved in BKPyV infection and cellular replication and its activation, which could be involved in cell transformation.

## 1. Introduction

BK polyomavirus (BKPyV) is a nonenveloped virus with an icosahedral structure containing a circular double-stranded DNA genome of ≃5 kb. It belongs to the *Polyomaviridae* family, genus polyomavirus. The virus is ubiquitous in the human population. It is typically contracted during childhood, with a seroprevalence of 65–90% in 5–9-year-old children [1]. After primary infection, BKPyV establishes a lifelong latency within the urogenital tract [2]. Under different circumstances, such as the use of immunosuppressive drugs, BKPyV can be reactivated, and it can be responsible for a series of clinical syndromes: allograft rejection, ureteral stenosis, hemorrhagic cystitis, nephropathy, and cancer [3, 4].

In the context of cell transformation, several studies evidenced the presence of BKPyV genetic material within various types of tumors, such as brain tumors, osteosarcomas, Ewing's tumors, neuroblastomas, and genitourinary tract tissue tumors, including prostatic and bladder cancers [5–9]. At the molecular level, it has been suggested that the BKPyV transforming activity depends on the early region of its genome, which encodes two viral oncoproteins: the large T-antigen (TAg) and the small t-antigen (tAg). TAg and tAg could be involved in alterations of the normal cell cycle resulting in cell immortalization and neoplastic transformation [10]. The most known process occurring under BKPyV infection is the interaction between BKPyV TAg and p53, which results in p53 inactivation. Consequently, the DNA damage response is altered, inducing the unscheduled onset of the S phase and leading to casual mutations that could activate cellular oncogenes or inhibit tumor suppressor genes [11]. However, other mechanisms through which BKPyV induces cell transformation are mostly neglected and require deeper studies.

An important aspect bridging virus infection and cell transformation is the alteration of the redox state toward the oxidative condition that in the former case is fundamental for viral replication, while in the latter for sustaining higher proliferation rate. We demonstrated that during different virus infections (e.g., herpes simplex virus type 1 (HSV-1), human immunodeficiency virus (HIV), and influenza virus), the content of glutathione (GSH), the most important nonenzymatic antioxidant, was diminished with a concomitant rise in oxygen/nitrogen reactive species (ROS/RNS) [12–14]. For some viruses, we found that such GSH decrement was pivotal not only for infection but also for viral protein folding and virus yield [15, 16]. In fact, using chemical compounds able to modulate intracellular GSH content, an inverse correlation was clearly demonstrated between the tripeptide content and infectivity [15, 17].

Cancer cells exploit higher radical flux for the increased proliferation rate and/or altered antioxidant systems. Indeed, ROS are increased in malignant cells in part as a result of oncogene signaling via the NADPH oxidase complex and by hypoxia-related mitochondrial ROS. Increased oxidant levels contribute to enhanced cell proliferation and apoptosis suppression [18]. At the same time, cancer cells required redox-mediated pathway adaptation in order to maintain cellular homeostasis under augmented ROS flux. Among such systems, innovative technologies have enabled the identification of various heat shock proteins (Hsps) as tumor markers [19]. Hsps are highly conserved proteins that can be activated following environmental or physiological stimuli, including infections. Hsp synthesis is tightly regulated at the transcriptional level by heat shock factor 1 (HSF1), which is considered the main regulator of the short-term induction of heat stress, and its activation/expression is used as a prognostic marker for a wide variety of cancers including kidney carcinoma [20–22]. Many oncoproteins require the high expression of Hsps to maintain their function; consequently, the protein levels of Hsps are significantly higher in a wide range of cancer cells than in normal cells. Hsps play a pivotal role in the progression of tumors and resistance against anticancer treatment and are currently considered therapeutic targets [23]. Among Hsps, Hsp70 and Hsp90 are reportedly correlated with the suppression of cell apoptosis and the promotion of cell proliferation and migration [24, 25]. Moreover, Hsps can be activated by redox imbalance. We previously demonstrated that the increase of the Hsp70 and Hsp27 expression levels, in addition to HSF1 activation, efficiently buffered the redox events downstream of chemical GSH depletion leading to an antiapoptotic action [26].

On the basis of this knowledge, in the present work, we characterized the effects of BKPyV infection in human embryonic kidney 293 (Hek293) cells, monitoring changes on intracellular redox environment, focusing on intracellular GSH and ROS/RNS production. Moreover, we demonstrated an increase of Hek293 cell proliferation under BKPyV replication that was associated with activation of the redox-sensitive HSF1-Hsp27 molecular axis, indicating these chaperones as fundamental early factors involved in neoplastic transformation following BKPyV infection.

## 2. Materials and Methods

### 2.1. Cell Culture and Treatments

Human embryonic kidney 293 (Hek293) cells were acquired from the European Collection of Cell Cultures (Salisbury, UK) and grown in DMEM with 10% FBS (Lonza, Basel, CH), 2 mM glutamine, and 100 U/ml penicillin/streptomycin and maintained at 37°C in a 5% CO_2_ atmosphere. Cells were plated in 6-well culture plates (1 × 10^6^ cells/well in 3 ml of DMEM with 10% FBS) and incubated at 37°C for 24 h.

Buthionine sulfoximine (BSO), a highly selective and potent inhibitor of the enzyme *γ*-glutamylcysteine synthetase (*γ*-GCS), was added in culture medium at a concentration of 1 mM. Quercetin (Q), an inhibitor of HSF1 [27], was added in culture medium at a concentration of 100 *μ*M.

### 2.2. Viral Propagation and Viral Titration

BKPyV was isolated from a positive urine sample of a patient admitted to Polyclinic Tor Vergata Foundation, Rome, Italy. To isolate infectious viral particles and produce the viral stock for experiments, confluent monolayers of Hek293 cells were incubated with the BKPyV positive urine sample, after filtration with 0.22 filter, for 1 h at 37°C in a 5% CO_2_ atmosphere. After the viral adsorption, the cells were washed with phosphate-buffered saline (PBS) and then incubated with medium supplemented with 2% FBS for 24 h. Viral titration was performed in the supernatants of infected cells 24 h postinfection using the BKPyV ELITe MGB® Kit according to the manufacturer's instruction. Total DNA was extracted from the supernatant by using the QIAsymphony DSP Virus/Pathogen Mini Kit from QIAGEN according to the manufacturer's instruction (QIAGEN S.p.A., Milan, Italy). BKPyV stock containing 250 copies/ml (cp/ml) was prepared and stored at -80°C.

### 2.3. Cell Infection and Treatments

Hek293 cells were plated, grown for 24 h, and then challenged with BKPyV stock diluted in serum-free medium. The clinical sample employed in this study was a residual specimen obtained in the course of institutional BKPyV diagnostic service.

BSO (1 mM) and Q (100 *μ*M) were added in culture medium 18 h before BKPyV infection and maintained throughout the experiment (24, 48, and 72 h). Cells were incubated in the presence of the virus for 1 h at 37°C in a 5% CO_2_ atmosphere. After the viral challenge, mock-infected and virus-infected cells were washed with PBS and then cultured with fresh medium containing 2% FBS, BSO, or Q.

### 2.4. Transfection with shRNA

shHSF1 (sequence: CGGCCAGCAACAGAAAGTCGTCAACTCGAGTTGACGACTTTCTGTTGCTGGTTTTT Sigma TRCN0000007482) was transfected by electroporation using Nucleofector 4D (Lonza, Sales) as previously described [28]. Moreover, Hek293 cells were transfected with siRNA duplex directed against the human p53 (SASI_Hs02_00302766) (sip53) target sequence. Transfection with a scrambled siRNA duplex (scr), with no homology to other human mRNAs, was used as control.

### 2.5. RT-qPCR Analysis

Total RNA was extracted using the TRI reagent (Sigma-Aldrich, St. Louis, MO). Three micrograms of RNA were used for retrotranscription with M-MLV (Promega, Madison, WI). qPCR was performed in triplicates by using validated qPCR primers (nucleotide BLAST, https://blast.ncbi.nlm.nih.gov), Ex TAq qPCR Premix (Lonza Sales, Basel, Switzerland), and the Real-Time PCR LightCycler II (Roche Diagnostics, Indianapolis, IN). mRNA levels were normalized vs. actin mRNA level, and the relative mRNA levels were determined by using the 2^−*ΔΔ*Ct^ method. The primer sequences are listed in Table 1.

### 2.6. Analysis of Cell Viability and Proliferation

Adherent (after trypsinization) and detached cells were combined, washed with PBS, and directly counted by an optical microscope on a hemocytometer, after Trypan blue staining. Cell proliferation was also assayed by a “Cell Proliferation kit” (Buckinghamshire, UK) based on the immunocytochemical detection of 5-bromo-2′-deoxyuridine (BrdU) incorporated into cellular DNA of proliferating cells. Cells were stained as previously described [29].

### 2.7. Preparation of Cell Lysates and Western Blot Analyses

Cell pellets were lysed in RIPA buffer (50 mM Tris-HCl, pH 8.0, 150 mM NaCl, 12 mM deoxycholic acid, 0.5% Nonidet P-40, and protease inhibitors). Protein samples were used for SDS-PAGE followed by Western blotting as previously described [30]. Nitrocellulose membranes were stained with primary antibodies against Tubulin (1 : 1000), p53 (1 : 1000), HSF1 (1 : 1000), Hsp27 (1 : 1000), Hsp90 (1 : 1000), and p-p38 (Santa Cruz Biotechnology, Dallas, TX). The nitrocellulose membranes were incubated with the appropriate horseradish peroxidase-conjugated secondary antibody (Bio-Rad), and immunoreactive bands were detected by a Fluorchem Imaging System upon staining with the ECL Select Western Blotting Detection Reagent (GE Healthcare, Pittsburgh, PA, USA; RPN2235). The Western blots reported are from one experiment out of three different experiments that gave similar results.

Protein content was assayed by the method described by Lowry et al. [31].

### 2.8. Determination of GSH

Intracellular GSH was assayed upon formation of S-carboxymethyl derivatives of the free thiol with iodoacetic acid, followed by the conversion of free amino groups to 2,4-dinitrophenyl derivatives by the reaction with 1-fluoro-2,4-dinitrobenzene and quantified through high-performance liquid chromatography (HPLC) as previously described [32].

### 2.9. Measurement of NOx

Accumulation of NOx in culture medium was measured by the Griess reaction, as previously described [33].

### 2.10. Evaluation of ROS Content

ROS were detected by cytofluorimetric analysis after incubation for 30 min at 37°C with 50 *μ*M H2DCF-DA as previously reported [34]. The fluorescence intensity of 10 000 cells from each sample was analyzed by the FACSCalibur instrument (Beckton-Dickinson, San Jose, CA, USA). Data were analyzed using the WinMDI 2.8 software (Scripps Research Institute, La Jolla, CA, USA).

### 2.11. Biotin Switch Assay

The Biotin Switch Assay was performed as previously described [28]. Proteins were subjected to S-NO derivatization by incubation with ascorbate. The negative control was made up of the same samples incubated in the presence of biotin without ascorbate. After protein separation by nonreducing SDS-PAGE and Western blotting, biotinylated proteins were detected by incubation of nitrocellulose membrane with HRP-conjugated streptavidin (1 : 1000).

### 2.12. Statistical Analyses

The results are presented as means ± S.D. Statistical evaluation was conducted as previously reported [35] by ANOVA, followed by the post-Student-Newman-Keuls or, when the samples to be analyzed were 2, the *t*-Student. Differences were considered to be significant at *p* < 0.05. All the data are from three biological replicates unless otherwise indicated in the figure legend.

## 3. Results

### 3.1. BKPyV Infection Relies on p53 in Human Hek293 Cells

**Figure 1:**
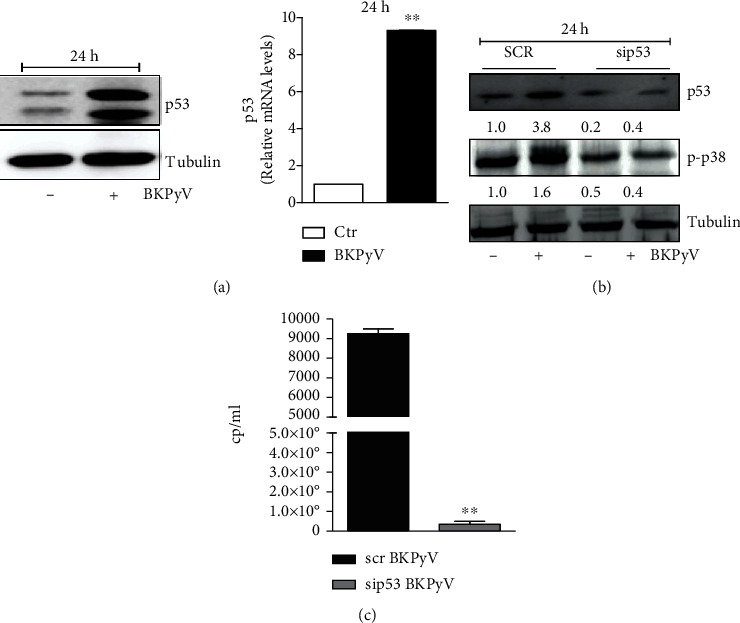
BKPyV infection requires p53. (a) Hek293 cells were infected with BKPyV for 1 h. Total protein extracts (20 *μ*g) were loaded for detection of p53 by Western blot. Tubulin was used as loading control (left panel). Total RNA was isolated, and relative mRNA level of p53 was analyzed by RT-qPCR (right panel). Data are expressed as means ± S.D. (*n* = 6, ^∗∗^*p* < 0.001). (b) Hek293 cells were transfected with scrambled (SCR) or p53 (sip53) siRNA. After 24 h, SCR and sip53 cells were infected with BKPyV for 1 h. Total protein extracts (20 *μ*g) were loaded for detection of p53 and p-p38 by Western blot. Tubulin was used as loading control. Densitometric analysis of p53 and p-p38 bands normalized for the loading control is reported below the corresponding band. (c) Viral titration was determined in the supernatants of infected cells 24 h postinfection using the BKPyV ELITe MGB® Kit (*n* = 5, ^∗∗^*p* < 0.001).

Although in literature the molecular mechanisms that drive BKPyV infection are not deeply outlined, a role for the tumor suppressor protein p53 in viral replication has been documented. In fact, it was demonstrated that p53 binds specifically to the viral promoter at two different sites and that the interaction between BKPyV TAg and p53 is fundamental for the virus replicative cycle [36–38]. Thus, to confirm the involvement of p53 under our experimental procedures, we infected human Hek293 embryonic kidney cell monolayer with 1 mml of BKPyV stock (250 cp/ml). After 1 h of viral adsorption, cells were washed and incubated with fresh medium for 24 h. Western blot analysis and RT-qPCR of p53 show that BKPyV infection dramatically increases the p53 expression at both protein and mRNA levels (Figure 1(a)). Subsequently, we confirmed the prominent role of p53 in the BKPyV replicative cycle by performing experiments upon p53 RNA interference (RNAi).  Figure 1(b) shows that p53 was efficiently downregulated in Hek293 cells by RNAi, and this resulted in complete inhibition of BKPyV replication (Figure 1(c)). The role of p53 in the process was emphasized by the lack of induction of mitogen-activated protein kinase, p38, a molecular factor deeply involved in the replicative cycle of different viruses [39]. In fact, Figure 1(b) shows that the phosphorylated active form of this protein was not significantly affected by BKPyV infection, under both resting and p53 interference.

### 3.2. BKPyV Infection Modifies Intracellular Redox State and Induces an Increase of RNS/ROS in Human Hek293 Cells

**Figure 2:**
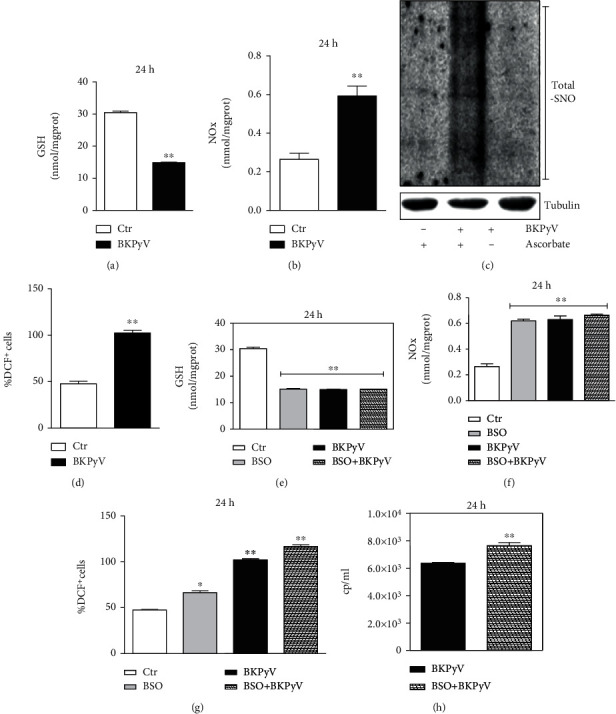
BKPyV infection results in RNS/ROS increase. Hek293 cells were infected with BKPyV for 1 h and analyses performed 24 h postinfection. (a) Intracellular GSH content was measured by HPLC. Data are expressed as means ± S.D. (*n* = 3; ^∗∗^*p* < 0.001). (b) Nitrite *plus* nitrate (NOx) in the culture medium was determined by Griess reaction. Data are expressed as means ± S.D. (*n* = 3; ^∗∗^*p* < 0.001). (c) Total proteins were subjected to S-NO derivatization with biotin. Tubulin was used as loading control. (d) ROS increase was evaluated by measuring DCF fluorescence by cytofluorimetric analysis. Data are reported as a percentage of DCF^+^cells ± S.D. (*n* = 3; ^∗∗^*p* < 0.001). (e–h) BSO (1 mM) was added in culture medium 18 h before 1 h BKPyV infection and maintained throughout the experiment for 24 h. (e) Intracellular GSH content was measured by HPLC. Data are expressed as means ± S.D. (*n* = 4; ^∗∗^*p* < 0.001). (f) Nitrite *plus* nitrate (NOx) in the culture medium was determined by Griess reaction. Data are expressed as means ± S.D. (*n* = 3; ^∗∗^*p* < 0.001). (g) ROS increase was evaluated by measuring DCF fluorescence by cytofluorimetric analysis. Data are reported as a percentage of DCF^+^cells ± S.D. (*n* = 3; ^∗^*p* < 0.05; ^∗∗^*p* < 0.001). (h) Viral titration was determined in the supernatants of infected cells using the BKPyV ELITe MGB® Kit (*n* = 6; ^∗∗^*p* < 0.001).

It has been established that one of the key events linked to viral infection is the modulation of the intracellular redox state. Indeed, an increase in the ROS/RNS level and/or the decrease in intracellular radical scavengers lead to the induction of oxidative/nitrosative stress, which is essential for replication of various viruses and the activation of the immune response and inflammation [40, 41]. Thus, to determine whether BKPyV infection was also accompanied by an oxidative imbalance, we measured the level of GSH, the most important low molecular weight antioxidant, frequently affected by infections.  Figure 2(a) shows a significant GSH depletion at 24 h after BKPyV infection compared to control cells, whereas no change in its oxidation form GSSG was determined (data not shown). Then, we analyzed whether such decrement was paralleled by an increase in radical species. To this aim, we determined the levels of nitric oxide (NO), by measuring in culture medium nitrites and nitrates (NOx), which represent the final NO-derived stable products.  Figure 2(b) shows that BKPyV infection leads to a significant accumulation of NOx at 24 h upon infection. This result was validated by analyzing, through the biotin switch assay, the content of S-nitrosylated proteins, one of the main intracellular targets of NO [42].  Figure 2(c) shows that BKPyV infection was also characterized by an increase in S-nitrosylated proteins with respect to noninfected cells. Finally, we measured intracellular ROS levels by means of cytofluorimetric analysis using the ROS-sensitive probe DCF-DA. We found that ROS were also significantly increased after BKPyV infection (Figure 2(d)). Overall, the data confirm that BKPyV infection resembles the behavior of other viruses in terms of intracellular redox state changes by both reducing antioxidants and increasing the production of radical species. With the aim to discern whether intracellular redox environment affects the extent of virus replication, we lowered the GSH content, before infection, by treating with buthionine sulfoximine (BSO), an irreversible inhibitor of *γ*-glutamylcysteine synthetase, the rate-limiting enzyme for GSH synthesis. 1 mM BSO was added in the culture medium of Hek293 cells 18 h before BKPyV infection and maintained throughout the experiments.  Figure 2(e) shows that BSO induced a significant decrease of intracellular GSH content that was similar to the decrement obtained upon BKPyV infection at 24 h, with no further decrease in the combined treatment. This result was mirrored by the increase of NOx levels (Figure 2(f)), while a synergic effect was observed for ROS content where the highest value was obtained after BSO treatment and BKPyV infection (Figure 2(g)). These changes finally resulted in a significant increase in virus yield, assessed through RT-qPCR, in BSO-treated cells (Figure 2(h)).

### 3.3. BKPyV Virus Infection Modulates Human Hek293 Cell Proliferation

**Figure 3:**
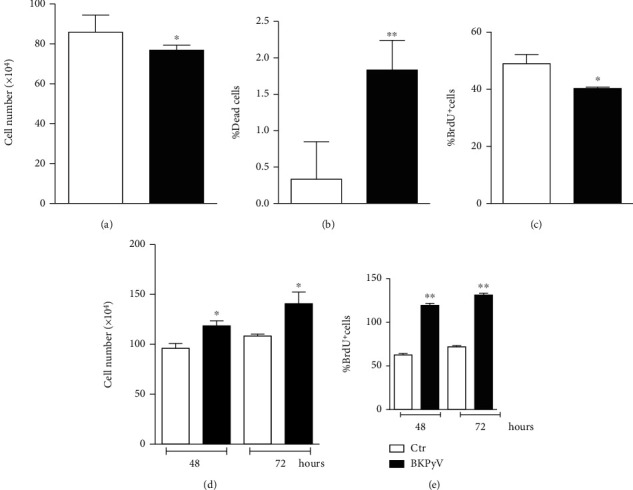
BKPyV induces Hek293 cell proliferation at late time of infection. Hek293 cells were infected with BKPyV for 1 h. (a, b) Cells were counted by Trypan blue exclusion after 24 h. Data are expressed as means ± S.D. (*n* = 3; ^∗^*p* < 0.05; ^∗∗^*p* < 0.001). (c) Cell proliferation was determined through immunofluorescence detection of incorporated BrdU. Data are expressed as means ± S.D. (*n* = 4; ^∗^*p* < 0.05). Hek293 cells were infected with BKPyV for 1 h. (d) Cells were counted by Trypan blue exclusion. Data are expressed as means ± S.D. (*n* = 6; ^∗^*p* < 0.05). (e) Cell proliferation was determined through immunofluorescence detection of incorporated BrdU. Data are expressed as means ± S.D. (*n* = 4; ^∗∗^*p* < 0.001).

It has been demonstrated that members of the *Polyomaviridae* family have the capacity of inducing cell transformation and tumorigenesis in different cell types, suggesting an etiological relationship with cancer [43]. In order to characterize new molecular mechanisms linking BKPyV infection with cell transformation, we investigated the effect of BKPyV on the proliferation of human Hek293 cells. After 1 h of viral adsorption, cells were incubated with fresh medium at different time points, and cell growth/viability was determined by direct count upon Trypan blue staining. We found that BKPyV infection at 24 h resulted in a decrement of cell number (Figure 3(a)) with a dissimilar increase in the number of dead cells (Figure 3(b)), suggesting that also proliferation arrest occurred. This was confirmed by anti-BrdU immunocytochemistry, which demonstrated a decrease in BrdU incorporation in BKPyV-infected cells after 24 h, indicative of cell cycle arrest in the G1 phase (Figure 3(c)). However, a completely different response was observed at longer time points, i.e., 48 and 72 h upon infection. As shown in Figure 3(d), the number of cells was significantly higher after both 48 and 72 h of BKPyV infection. The cell proliferation increase was time-dependent (Figure 3(d)) and without changes in the number of Trypan blue positive cells (data not shown). Anti-BrdU immunohistochemistry was then employed to confirm the data obtained by the Trypan blue assay.  Figure 3(e) shows that after 48 and 72 h of BKPyV infection, approximately 55% of cells incorporated BrdU (cells in S phase), confirming that BKPyV promotes Hek293 cell proliferation at late times of infection.

### 3.4. BKPyV-Induced Proliferation Was Associated with HSF1 Activation in Human Hek293 Cells

**Figure 4:**
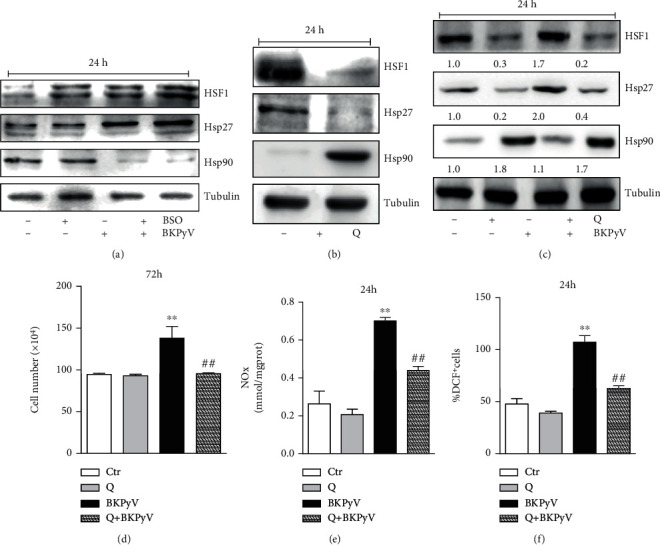
BKPyV infection affects HSF1-Hsp27 signaling pathway. BSO (1 mM) or Q (100 *μ*M) was added in culture medium 18 h before 1 h BKPyV infection and maintained throughout the experiment for the indicated time. (a–c) Cells were lysed, and total protein extracts (20 *μ*g) were loaded for detection of HSF1, Hsp27, and Hsp90 by Western blot. Tubulin was used as loading control. Densitometric analysis of (c) is reported below the corresponding band. (d) Cells were counted by Trypan blue exclusion. Data are expressed as means ± S.D. (*n* = 4; ^∗∗^*p* < 0.001*vs.* Ctr cells; ^##^*p* < 0.001*vs.* BKPyV-infected cells). (e) Nitrite *plus* nitrate (NOx) in the culture medium was determined by Griess reaction. Data are expressed as means ± S.D. (*n* = 3; ^∗∗^*p* < 0.001*vs.* Ctr cells; ^##^*p* < 0.001*vs.* BKPyV-infected cells). (f) ROS increase was evaluated by measuring DCF fluorescence by cytofluorimetric analysis. Data are reported as a percentage of DCF^+^cells ± S.D. (*n* = 4; ^∗∗^*p* < 0.001*vs.* Ctr cells; ^##^*p* < 0.001*vs.* BKPyV-infected cells).

We previously reported that cell response to GSH depletion involves induction of heat shock proteins, such as Hsp27 and Hsp70, which dampen oxidative stress-induced death by counteracting apoptosis [44–46]. The upstream key player in cellular stress response is HSF1, which activates important signaling pathways to maintain cellular homeostasis, a function that is overlapping with that of p53. Moreover, HSF1 is mainly activated by proteotoxic stress, a process that characterizes viral replication [47, 48]. This evidence prompted us to investigate the possible involvement of HSF1 in the increased proliferation of Hek293 cells upon BKPyV infection in relation to the redox state. To this purpose, we utilized BSO as a glutathione synthesis inhibitor able to induce a mild oxidative environment. 1 mM BSO was added in the culture medium of Hek293 cells 18 h before BKPyV infection and maintained throughout the time course of the experiments. Subsequently, we performed a Western blot analysis of chaperonins starting from the upstream transcription factor HSF1. As reported in Figure 4(a), HSF1 was increased upon BKPyV infection at 24 h more efficiently with respect to BSO treatment. Moreover, Figure 4(a) shows a decrease of Hsp90 mainly under BKPyV infection proposing that dissociation between the two proteins occurred more efficiently upon BKPyV infection, leading to HSF1 activation. Indeed, under physiological conditions, HSF1 is normally present in the cytoplasm as a dephosphorylated/inactive form by binding to Hsp90. Following stress, Hsp90 interacts with unfolded proteins and releases HSF1, which translocates into the nucleus, trimerizes, and binds sequences of DNA known as heat shock elements (HSE), leading to transcription of Hsp genes [49]. Figure 4(a) shows a significant increase in the expression levels of Hsp27, confirming a role for HSF1 activation and signaling in BKPyV infection.

To deeply investigate the role played by the HSF1-Hsp27 pathway in BKPyV-induced Hek293 cell proliferation, we assessed the effect of quercetin (Q), a bioflavonoid that inhibits HSF1 by specifically blocking HSF1 trimerization and nuclear translocation [50]. First, we tested the efficacy of 100 *μ*M Q in inhibiting HSF1, and, as showed in Figure 4(b), this compound efficiently modulated the signaling pathway in terms of Hsp27 and Hsp90 expression levels. Subsequently, Q was added in culture medium 18 h before BKPyV infection and maintained for the following 24 h. Western blot analysis reported in Figure 4(c) indicates that, also in BKPyV-infected cells, Q caused a significant reduction of HSF1 and Hsp27 protein levels accompanied by an increase in the levels of Hsp90. Then, we evaluated the effect of HSF1 inhibition on Hek293 cell proliferation. Q significantly inhibited BKPyV-mediated proliferation at 72 h, as assayed by cell counting (Figure 4(d)). This was associated with a strong inhibition of NOx released in the culture medium and intracellular ROS accumulation after 24 h of BKPyV infection (Figures 4(e) and 4(f), respectively).

**Figure 5:**
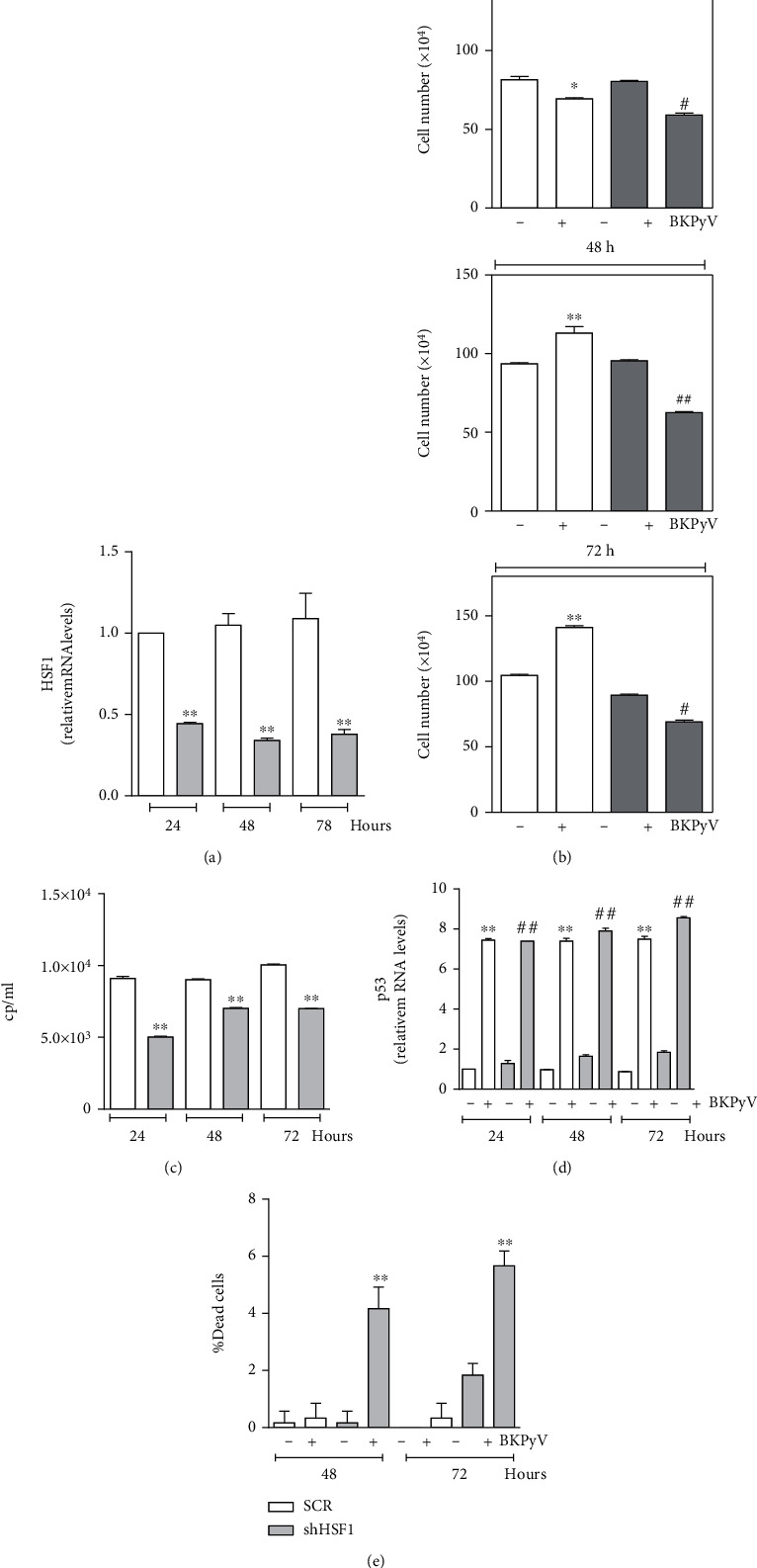
HSF1 activation is involved in increased Hek293 cell proliferation upon BKPyV infection. Hek293 cells were transfected with scramble (SCR cells) or shHSF1 (shHSF1 cells) and after 24 h cells were infected with BKPyV for 1 h. (a) Total RNA was isolated, and the relative mRNA level of HSF1 was analyzed by RT-qPCR. Data are expressed as means ± S.D. (*n* = 4, ^∗∗^*p* < 0.001). (b) Cells were counted by Trypan blue exclusion. Data are expressed as means ± S.D. (*n* = 3; ^∗^*p* < 0.05 or ^∗∗^*p* < 0.001*vs.* SCR cells; ^#^*p* < 0.05 or ^##^*p* < 0.001*vs.* shHSF1 cells). (c) Viral titration was determined in the supernatants of infected cells using the BKPyV ELITe MGB® Kit (*n* = 6; ^∗∗^*p* < 0.001). (d) Total RNA was isolated, and the relative mRNA level of p53 was analyzed by RT-qPCR. Data are expressed as means ± S.D. (*n* = 6, ^∗∗^*p* < 0.001*vs.* SCR; ^##^*p* < 0.001*vs.* shHSF1). (e) Cells were counted by Trypan blue exclusion. Data are expressed as means ± S.D. (*n* = 3; ^∗∗^*p* < 0.001).

Since Q is a polyphenol antioxidant, it could be responsible for a wider range of effects on cell homeostasis, including buffering of radical species, a process that was efficiently detected in our experimental system. Therefore, we further investigated the role played by the HSF1-Hsp27 pathway by specifically downregulating HSF1 through a short hairpin RNA. HSF1 downregulation was efficiently achieved in Hek293 cells at 24 h and maintained at 48 and 72 h (Figure 5(a)). As observed with Q, lowering the HSF1 level efficiently inhibited cell proliferation upon BKPyV infection both at 48 and 72 h (Figure 5(b)). It is also worth noting that at 72 h, the downregulation of HSF1 induces a slight inhibition also in the proliferation of the control cells. Then, we assayed the release of viral particles from Hek293 cells through the RT-qPCR analysis. We found that viral copies were reduced in shHSF1 cells (Figure 5(c)) but not completely abolished. Moreover, virus titer increased between 24 and 48 h, suggesting a still active virus replication process.

Finally, to better understand the specific role of p53 and HSF1 in our experimental system, we analyzed the expression of p53 upon downregulation of HSF1.  Figure 5(d) shows an increase of p53 expression following viral infection not only in SCR cells but also in shHSF1 cells. These results indicate that while p53 is involved in viral replication, HSF1 is instead deeply involved in regulating the Hek293 proliferation rate. Under this experimental condition, we observed that the inhibition of cell proliferation and a still active virus replication result in a cytopathic effect at later time points, as reported in Figure 5(e), where a significant number of cell death were determined.

## 4. Discussion

Several studies show a relationship between BKPyV infection and urothelial or kidney carcinoma cells in renal allograft recipients, affecting either the transplanted organ or the genitourinary tract of the recipient [51–53]. This evidence, in correlation with the knowledge that the kidney is the latency site of BKPyV [54], suggests that urinary tract cancer could represent the best candidate for an etiological association with BKPyV. Understanding the molecular mechanism underlying tumor cell proliferation is of major importance to develop more precise and noninvasive antikidney cancer therapies.

In this work, we have highlighted a new molecular pathway activated by BKPyV infection in kidney epithelial cells suggesting the redox-mediated HFS1-Hsp27 axis as responsible for increased cell growth. The two-step process observed in the proliferation of Hek293 cells, with virus-induced cell cycle arrest at 24 h and the increased proliferation rate at 48/72 h, suggests an efficient interference put in place by the virus in the replicative system of the host cell. Our results, at 24 h of infection, are in agreement with data present in the literature showing that infectious bronchitis virus (IBV) induces a G2/M-phase arrest in infected cells to favor progeny virus production [55]. Indeed, many DNA viruses have evolved mechanisms to override normal checkpoint control and force cells into the S phase to ensure an abundant supply of nucleotides and other essential replication factors during their replication cycles [56].

Oxidative and/or nitrosative stress has been reported to occur in several different viral infections both *in vivo* (e.g., influenza [57]) and *in vitro* (e.g., parainfluenza and herpes simplex type 1 [13]), and it seems to be a common feature characterizing the replicative cycle of different viruses [12, 14, 17]. In this context, GSH plays an important role as a ROS/RNS buffer and as a guardian of intracellular redox environment avoiding damage to the macromolecules [58, 59]. The data obtained with BKPyV mirrored those previously obtained with other viruses, as we determined GSH depletion and increased radical flux. The importance of an increased oxidative environment in the replicative cycle of BKPyV was also highlighted by the treatment with BSO that demonstrated a direct correlation between radical flux and virus yield.

Data from the literature show that the oncogenicity of BKPyV is given by the ability of its TAg protein to interact with p53 [5, 60]. p53 regulates the checkpoint between G1 and S phases of the cell cycle especially in the presence of DNA damage [61]. It was suggested that the interaction of TAg with p53 resulted in altered p53 functions, one of which could be the failure to function as a guardian of cell cycle control [62]. Moreover, p53 has been demonstrated to bind the early viral promoter of BKPyV DNA modulating the expression of BKPyV oncoproteins [37]. Here, we recapitulated the prominent role of p53 in BKPyV replication as p53 RNA interference completely abrogated virus production.

On the other hand, the increased proliferation rate observed in Hek293 cells at longer times of infection was strictly dependent on HSF1 activation and downstream synthesis of Hsp27. HSF1 and its gene targets are essential for tumorigenesis across several experimental tumor models and facilitate metastatic and resistant properties within cancer cells. HSF1 activity is augmented in many tumor contexts in a way that resembles a chronic state of stress, characterized by high levels of HSP gene expression [63]. For instance, Hsp27 is involved in tumor proliferation by activating the transcription of AP1, which modulates the expression of cell cycle regulators such as p53, p21, Cyclin D1 [64, 65]. It is also involved in the inhibition of apoptosis and drug resistance [66–68], whereas HSF1 regulates tumor progression during the metastatic process and is used as a biomarker for tumors that are progressing toward metastasis [69, 70]. In our experimental system, by using an RNA short hairpin for HSF1, we observed a complete inhibition of cell proliferation at 48 and 72 h of infection, with a significant reduction in virus yield, but not a complete inhibition as observed under p53 interference. The virus was able to replicate under HSF1 downregulation, and the inhibition in Hek293 cell proliferation results in an increased cytopathic effect leading to cell death. The evidence of active virus replication was also confirmed by the high expression levels of p53 determined under HSF1 downregulation.

Finally, the data obtained with quercetin were highly important not only for validating the role played by HSF1-Hsp27 in BKPyV-induced Hek293 cell proliferation but mostly for clinical translation. Quercetin is widely present in nature in fruits, seeds, flowers, and leaves, as well as in medicinal plants, such as Ginkgo biloba and Hypericum perforatum [71]. Its molecular structure contains active groups among which the presence of a phenolic hydroxyl group and double bonds endows quercetin with strong antioxidant activity [72]. Its antioxidant and anti-inflammatory properties are closely related to therapeutic activities against various diseases [72, 73]. Our data clearly showed an efficient inhibitory effect of quercetin in the HSF1-signaling pathway resulting in significant inhibition of BKPyV-induced Hek293 cell proliferation as well as in the reduction of ROS/RNS intracellular content. Quercetin being a safe natural antioxidant can be used in animal feed for *in vivo* experiments of BKPyV viral replication.

In conclusion, the data obtained in this work evidenced HSF1-Hsp27 as new molecular factors involved in BKPyV infection and replication, which could be responsible for inducing cell transformation. Overall, the pathological conditions that characterize a tumor-initiating cell phenotype require growth signals and evasion of death pathways, as well as the modulation of metabolic programming. These aspects are highly linked to HSF1 biological involvement in tumorigenesis and give efforts to the hypothesis of BKPyV-induced transformation. At the same time, the role highlighted for quercetin in counteracting BKPyV replication and cell proliferation suggests a new avenue for therapeutic interventions.

## Figures and Tables

**Table 1:** List of primers used for reverse transcription-quantitative polymerase chain reaction.

Name	FW	RV
p53	5′-GTGACACGCTTCCCTGGATT-3′	5′-CTCCGTCATGTGCTGTGACT-3′
HSF1	5′-GAAGCAGCTGGTGCACTACA-3′	5′-TATCTCTGTGGAGCGGGGAA-3′
Actin	5′-GATTCCTATGTGGGCGACGA-3′	5′-CGGACTCGTCATACTCCTGC-3′

## Data Availability

The data used to support the findings of this study are included within the article.
